# Using Probabilistic Movement Primitives in Analyzing Human Motion Differences Under Transcranial Current Stimulation

**DOI:** 10.3389/frobt.2021.721890

**Published:** 2021-09-14

**Authors:** Honghu Xue, Rebecca Herzog, Till M. Berger, Tobias Bäumer, Anne Weissbach, Elmar Rueckert

**Affiliations:** ^1^ Institute for Robotics and Cognitive Systems, University of Luebeck, Luebeck, Germany; ^2^ Institute of Systems Motor Science, University of Luebeck, Luebeck, Germany; ^3^ Department of Neurology, University Medical Center Schleswig-Holstein, Luebeck, Germany; ^4^ Institute of Neurogenetics, University of Luebeck, Luebeck, Germany; ^5^ Chair of Cyber-Physical-Systems, Montanuniversität Leoben, Leoben, Austria

**Keywords:** probabilistic movement primitives, human motion analysis, finger tapping motion, machine learning, transcranial current stimulation

## Abstract

In medical tasks such as human motion analysis, computer-aided auxiliary systems have become the preferred choice for human experts for their high efficiency. However, conventional approaches are typically based on user-defined features such as movement onset times, peak velocities, motion vectors, or frequency domain analyses. Such approaches entail careful data post-processing or specific domain knowledge to achieve a meaningful feature extraction. Besides, they are prone to noise and the manual-defined features could hardly be re-used for other analyses. In this paper, we proposed *probabilistic movement primitives* (ProMPs), a widely-used approach in robot skill learning, to model human motions. The benefit of ProMPs is that the features are directly learned from the data and ProMPs can capture important features describing the trajectory shape, which can easily be extended to other tasks. Distinct from previous research, where classification tasks are mostly investigated, we applied ProMPs together with a variant of Kullback-Leibler (KL) divergence to quantify the effect of different *transcranial current stimulation* methods on human motions. We presented an initial result with 10 participants. The results validate ProMPs as a robust and effective feature extractor for human motions.

## 1 Introduction

Human motor coordination has been extensively investigated in medical research (Rosenbaum, 2009) such as post-stroke rehabilitation (Gresham et al., 2004; Hatem et al., 2016), Parkinson (Plotnik et al., 2007; Inzelberg et al., 2008), alcoholism (Sullivan et al., 1995; Marczinski et al., 2012), and so on. One typical task is to examine the human motions with the presence of certain external stimuli to verify its effectiveness on human motor control. Prior to the existence of auxiliary analysis tools, examination of such effect relies on human expert via visual observation, which often results in low efficiency and less objectivity. For instance, in the case of fast movements, it would be hard for human experts to distinguish motions behavior, and high concentration easily gives rises to fatigue, consequentially incurring a less accurate diagnosis. On the other hand, an auxiliary analysis tool can significantly reduce the workload, increase the efficiency as well as the accuracy. In this work, we extend the method of *Probabilistic Movement Primitives* (ProMPs) (Paraschos et al., 2013), a well-established approach in robotics skill learning, to human motor coordination analysis.

One exploratory field in medical research is *noninvasive brain stimulation*, which has been long assumed as an alternative to treat neurological and psychiatric disorders. However, the brain stimulation technique is still a young research field and real applications are largely unexplored. One interesting topic is whether *noninvasive brain stimulation* can influence cerebellar excitability and connectivity, consequentially leading to the changes in human motor coordination. Among all brain stimulation methods, *transcranial current stimulation* (tCS) is gaining popularity due to its easy deployablility in application (Datta et al., 2008; Brittain et al., 2013; Filmer et al., 2014). In the family of tCS, *transcranial direct current stimulation* (tDCS) is one popular method (Nitsche et al., 2008; Caumo et al., 2012; Thair et al., 2017). However, tDCS shows a high variability of study protocols and applications, which registers heterogeneity of results. tDCS only exerts general effect on underlying neuron populations, without discriminating them. Therefore, other current stimulation methods serve as interesting alternatives, especially *transcranial alternating current stimulation* (tACS), whose effect has been previously studied on arm movements (Naro et al., 2016; Naro et al., 2017). Additionally, tACS can influence intrinsic oscillations, where tDCS has no influence. Besides tDCS and tACS, we also investigate the effect of *transcranial random noise stimulation* (tRNS) of the cerebellum. We examine how human motions are affected under these three variants of tCS conditions.

In this paper, we applied ProMPs to evaluate the effect of tDCS, tACS and tRNS on human arm motions. Specifically, we examined whether the different stimuli approaches influenced the motion patterns as well as the duration of stimulation effect. We recorded the arm motions by inertial measurement units (IMUs) mounted on hands and wrists similar to the approach in Krishna et al. (2019). We quantified the stimulation effects by measuring the motion difference between stimulated conditions and non-stimulated conditions in eight different finger-tapping experiments, where the influence on motion coordination could be present (Benussi et al., 2017; Orrù et al., 2020). In a finger-tapping experiment, the degree of freedom in finger tapping task is restricted, which greatly reduces the undesired effects not caused by stimulation, e.g., human exploratory behavior. The motion difference is evaluated by mapping the trajectories into feature space via ProMPs and then using a variant of *Kullback-Leibler divergence* (KL-divergence) as a distance metric. In this work, we first presented an initial result with 10 participants.

In summary, the main contributions of this paper are as follows: i) We proposed *probabilistic movement primitives* as an efficient and robust feature extractor characterizing human motions, where the features were completely learned from the dataset. ii) We quantified the effects of the different non-invasive brain stimulation approaches tACS, tDCS and tRNS using *symmetric Kullback-Leibler divergence* and *probabilistic movement primitives*. iii) We presented the initial results on 10 participants, showing our approach as an advantageous auxiliary analysis tool, and discussed the advantages and limitations. iv) We showed a complete workflow on data collection, data post-processing, coupled with details on how *Probabilistic Movement Primitives* fit on the data using IMUs.

### 2 Related Work

In this section, we review the previous approaches on analysing human motions and variants of *movement primitives* applied in robot skill learning and human motion modelling.

### 2.1 Human Motion Analyses

In order to measure the difference between the sets of trajectories, it is essential to capture some features of the trajectories, i.e., mapping the trajectory into a feature space. Several previous studies on analyzing motion difference depend either on manually-designed features of the trajectories (Bologna et al., 2016; Kwak et al., 2020; Marković et al., 2020) or on frequency-domain analysis (Omkar et al., 2011; Krishna et al., 2019).

In Bologna et al. (2016), they examined the effects of cerebellar theta-burst stimulation on patients with focal dystonia by quantifying the changes in arm and neck movements. They captured the neck/arm movements by IMUs. For the head movements, they extracted the features such as angular amplitude and the maximal angular velocity, while for arm movements, the trajectory straightness, the smoothness of arm velocity curves and target overshooting were analysed. Such kinematics data were subsequently analysed by Kruskar-Wallis analysis of variance (ANOVA). In Kwak et al. (2020), they aimed to measure the difference of motion smoothness between a nonpathologic shoulder and a shoulder with a rotator cuff tear. They characterized such difference by measuring the angular velocity using IMUs, where they manually defined the number of peaks, peak velocity peak velocity–to–mean velocity ratio, and the number of sign reversals. However, it is noteworthy that the authors need to pre-define a set of kinematic features to characterize the motion difference, which requires good domain knowledge and can hardly be extended to other different tasks. However, ProMPs could largely mitigate this issue. We will discuss these aspects in details in Section 6.

Another work (Punchihewa et al., 2020) examined the validity and reliability of IMUs in evaluating hand and trunk kinematics in a baseball-hitting scenario, the authors compared the performance between IMUs and optical motion capture systems by measuring the root mean square error across the angular displacement curves. In their paper, they also show how they derived the kinematic parameters from IMUs. The finding was that IMUs with the sampling rate of 1,000 *Hz* were sufficient in quantifying trunk and hand movement coordination in a hitting movement. Some similar findings of the efficacy of IMUs in measuring rapid movements was also verified in Marković et al. (2020), where they discriminated the hand tapping motions among three different groups of females featuring different age and jobs. The difference was measured through descriptive statistical analysis on features such as motion onset time, peak acceleration/deceleration and acceleration/deceleration gradients. Based on these previous studies, we also used IMUs to capture motions from hands and wrists for its easy deployability.

There are also a variety of other works to measure the human motion difference, for instance, vision-based approaches for action segmentation or so on (Wang et al., 2003; Chen et al., 2013). And lots of previous work mainly focused on classification tasks, where various classifiers such as neural networks (Hiraiwa et al., 1989; Steven Eyobu and Han, 2018), linear discriminant analysis (Krishna et al., 2019), and support vector machines (Groh et al., 2015; Li et al., 2016) were applied. They are normally coupled with some feature pre-processing techniques, e.g., principal component analysis. In this study, instead of classifying, we determined the degree of similarity between sets of trajectories numerically.

### 2.2 Movement Primitives

*Movement primitives* (MPs) (Schaal et al., 2005) have been extensively studied in robotics to model arbitrarily complex motor skills from both robots and humans by modularly executing multiple basic movement patterns sequentially or in parallel.

One popular approach in the family of MPs is *dynamic movement primitives* (DMPs) (Schaal et al., 2003; Schaal, 2006; Ijspeert et al., 2013). DMPs are mathematically characterized by a second-order damping system with an additional forcing term. The second-order damping system with a goal attractor asserts an asymptotic convergence to a desired pose at the end of the trajectory while the forcing term increases the model capacity to approximate trajectories of arbitrary shapes. DMPs have been widely used in robot learning, e.g., a robot pouring task (Tamosiunaite et al., 2011). In Prada et al. (2013), Umlauft et al. (2014), they applied DMPs in human-robot collaboration or interactions tasks, for instance, object hand-over. (Pervez and Lee, 2018) generalized DMPs from single-task learning to multiple tasks *task-parameterized DMPs*, where they jointly learned the probability distribution of task-specific parameters and the shape parameters of DMPs. When inferring the trajectory for an unseen task, the phase as well as the task-specific parameters will be passed. Some other extensions for DMPs focused on online adaptation, where one of the typical tasks could be collision avoidance (Park et al., 2008; Hoffmann et al., 2009; Tan et al., 2011).

While DMPs show great success in learning motor tasks, they only represent single elementary action. In contrast, *probabilistic movement primitives* (ProMPs) (Paraschos et al., 2018) model the trajectory in a probabilistic manner, which offers more flexibility than a deterministic model. Firstly, a probabilistic model can represent the motion uncertainty at every time point during the demonstrations. This uncertainty can be used to adapt control parameters (Paraschos et al., 2013). Furthermore, ProMPs can model the coupling between joints which is essential for controlling and modelling high-dimensional coupled kinematic chains such as humans and robots. A probabilistic characterization with a coupled relation also allows common probability operation such as conditioning, where a complete trajectory on all joints can be inferred from given via-points. In the case of inferring a trajectory from given via-points from different tasks, ProMPs outperforms DMPs in terms of accuracy at via-points and its adaptable variance (Paraschos et al., 2018). Similar to DMPs, ProMPs can also be extended to multiple tasks by appending task parameters to trajectory shape parameters and performing joint linear regression (Rueckert et al., 2015).

In robotic tasks, ProMPs can be fitted on a couple of demonstrations using imitation learning (Maeda et al., 2014; Gomez-Gonzalez et al., 2016). With a learned ProMP model, robots can either subsequently reproduce the demonstrated motion pattern or even improve the trajectory gradually via *trajectory optimization* (Tamosiunaite et al., 2011). Paraschos et al. (2018) verified the performance of ProMPs on benchmark tasks such as 7-link reaching task, robot hockey, playing table tennis and so on, especially in the task of table tennis, the performance of ProMPs is superior to DMPs, leading generally to smaller errors with an increased success rate. Other work also covered combining transfer learning with MP (Rueckert et al., 2015), where robots were trained on a few tasks and later generalize to unseen ones. (Ewerton et al., 2015; Maeda et al., 2017) further extended the idea of ProMPs to a Gaussian mixture of ProMPs for collaborative robots to coordinate the movements of a human partner.

### 2.3 Using Probabilistic Movement Primitives for Human Motion Analysis

With the success in modelling robot motions, it is promising to extend ProMPs to human motions. Several previous work has been extensively applying MPs to model human motor skills (Lin et al., 2016). The prior work (Rueckert et al., 2016) used ProMPs to analyze the human adaptation with the presence of external perturbations by investigating the correlation between the motions of both arms and the trunk. In this work, they showed that ProMPs could sufficiently predict the complete trajectory of the right arm only, given the trajectory of the left arm at initial phases. This finding showed that ProMPs achieve a reasonable performance of modelling the multiple trajectories in a coupled setting, which is highly-related to our work where we quantify the trajectory difference from a mixture of trajectories, i.e., from both hand and wrist. In another work (Kohlschuetter et al., 2016), they used ProMPs on EMG data to predict knee anomalies. By learning the prior distribution of the weights on the post-processed EMG data, they passed the weights of the features including both mean and variance to the classifier. They showed that a probabilistic model achieves a higher prediction accuracy than a deterministic model with no uncertainty measure. Our work is related to them in a way that we measured the trajectory difference in a probabilistic manner (with uncertainty measure), potentially being more accurate than a deterministic model.

Some other work also proposed using MPs for human motions. (Lim et al., 2005) proposed using MPs to generate natural, human-like motions with a framework combining dynamic models and optimization. For instance, (Rueckert and d’ Avella, 2013) suggested a movement primitive representation as a generalized case of DMPs to implement shared knowledge in the form of learned synergies, where the learned synergies summarized the muscle excitation patterns and enabled transferring to other tasks given muscle signal in musculoskeletal systems.

Based on the previous studies, we proposed using ProMPs for modelling the coupled movements from hand and wrist to determine the effect of *transcranial current stimulation*. To the best of our knowledge, this work is the first attempt to apply ProMPs to investigate effect on non-invasive brain stimulation approaches.

## 3 Methods

In this section, we provide a mathematical formulation on *Probabilistic Movement Primitives* (ProMPs) and subsequently show how to fit the motion trajectories using ProMPs. We start by introducing the definition of *time-series data*, on which ProMPs will be fitted. Afterwards, we extend a single time-step case to multi time-step case, i.e., a set of complete trajectories. Finally, we generalize a complete trajectory to coupled trajectories.

### 3.1 ProMPs as a Probabilistic Time Series Model

A *trajectory* *τ* is a sequence of observations *y*. Mathematically, we define a complete trajectory as ***τ***_***j***_ = [*y*_0,*j*_, …, *y*_*T*,*j*_], where *y*_*t*,*j*_ denotes the observed measurements at time point *t* and the trajectory *j* has the length of *T*. We assume scalar observations where $y_{t,j}\in R^{1}$ and later we will extend to multi-dimensional cases. Now, we consider a set of trajectories with the same length *T*, and arrive at the data form ***S*** = {*y*_0,1_, *y*_1,1_, …, *y*_*T*,*n*_}, where *n* refers to the number of demonstrations or trajectories. The goal is to derive a probabilistic time series model from these *n* trajectories denoted by *p*(***τ***).

### 3.2 Probabilistic Time Series Model on Single Time Step

We first start with a probabilistic characterization on ***S*** the for a single time step using Gaussian distribution. For simplicity, we use the notation ***y***_***t***_ to represent a set of *n* observations at time step *t*, i.e., $y_{t}=\left[\left[y_{t,1},y_{t,2},\ldots ,y_{t,n}\right]\right]\in R^{1\times n}$. We assume a Gaussian distribution on the observations with the mean as a linear combination of non-linear features *ϕ* and a fixed variance, shown as below:

$$P\left(y_{t}|w\right)=N\left(y_{t}|\phi_{t}w,\sigma_{y}^{2}\right),$$ (1)

where $\phi_{t}\in R^{1\times M}$ denotes the feature vector at the time step *t* and $w\in R^{M\times n}$ denotes the weight vector to be learned. In this case, we assume the number of used basis functions to be *M*. The standard deviation of the observations *σ*_*y*_ can be interpreted as a noise term.

**FIGURE 1:**
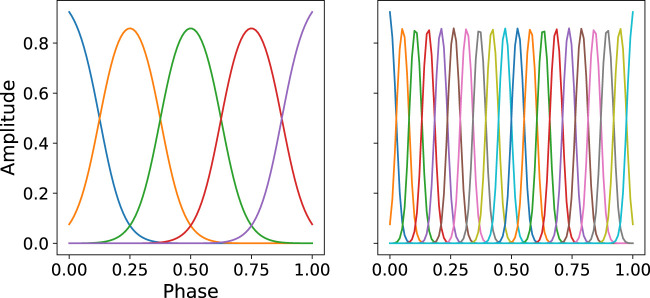
Illustration of the distribution of basis function. **Left:** *M* = 5, **Right:** *M* = 20. The bandwidth parameter is computed by $h=0.2\cdot {\left(c_{i+1}-c_{i}\right)}^{2}$ to ensure an overlap between neighboring basis functions.

The common choice of the feature $\phi_{t}^{i}\in R^{1}\;,\forall i\in \left[1,\ldots ,M\right]$ varies for stroke-based movements or rhythmic movements (Paraschos et al., 2013). In our case, the finger-tapping trajectory are firstly segmented to a set of point-to-point trajectories, where the stroke-based movements are appropriate choices, shown as follows:

$$\begin{array}{cc} \phi_{t}^{i} & =\exp\left(-\frac{{\left(z_{t}-c_{i}\right)}^{2}}{2h_{i}}\right),\\ \phi_{t} & =\frac{1}{\sum_{i=1}^{M}\phi_{t}^{i}}\left[\left[\phi_{t}^{1},\ldots ,\phi_{t}^{M}\right]\right], \end{array}$$ (2)

where *c*_*i*_ refers to the center and *h*_*i*_ refers to the bandwidth of the *i*-th basis function, $z_{t}=\frac{t}{T}\in \left[0,1\right]$ is the *movement phase*, which is a generalization of time that allows for generating motions of arbitrary duration. An illustration on how basis functions are spread over time is shown in Figure 1. Until now, we describe how to map measurements of single time step ***y***_***t***_ into a feature space *ϕ*. Then, we show how to compute the optimal ProMPs model parameters $w^{⋆}\in R^{M\times n}$ on that single time step data ***y***_***t***_. In principle, ***w*** ^***⋆***^ can be learned via *expectation maximization* (Rueckert et al., 2015) or simply through *damped least-square regression*, i.e., *ridge regression*.

$$w^{⋆}={\left(\phi_{t}^{T}\phi_{t}+\lambda I\right)}^{-1}\phi_{t}^{T}y_{t},$$ (3)

where ***I*** is the identity matrix and the additional term of *λ* avoids the singularities which can be caused by small or poorly-sampled datasets. The poorly-sampled data refers to the importance of motion variance in the data (copies of the same demonstrations would result in singular matrices).

### 3.3 Probabilistic Time Series Model on Multi-Time-Step Data and Multi-Dimensional Observations

The previous subsection describes how to learn the weights from multiple demonstrations on a single time step. In this part, we extend it to multi-time step setting, i.e., learning the weights from multiple complete trajectories. We assume the observations in each time step are *i. i.d.*.

$$\begin{array}{cc} p\left(\tau |w\right) & =p\left(y_{0},y_{1},\ldots ,y_{T}|w\right)\\ & =\overset{T}{\underset{t=0}{\prod }}p\left(y_{t}|w\right)\\ & =\overset{T}{\underset{t=0}{\prod }}N\left(y_{t}|\phi_{t}w,\sigma_{y}^{2}\right)\\ & =N\left(\tau |\Phi w,\sigma_{y}^{2}I\right), \end{array}$$ (4)

where the matrix $\Phi =\left[\phi_{0},\phi_{1},\ldots ,\phi_{T}\right]\in R^{T\times M}$. The optimal weights are computed in the same way as in Eq. 3 by replacing *ϕ*_***t***_ with **Φ**.

In the above parts, we introduce how to fit ProMPs on a set of trajectories in the case of scalar observations, where $y_{t,j}\in R^{1}$. Here, we extend the setting to multi-dimensional observations, i.e., $y_{t,j}\in R^{D}$ and $\tau \in R^{\left(T\cdot D\right)\times n}$, where *D* is the number of dimensions. In our case, we have a six-dimensional observation for each time-step, respectively *x*, *y* and *z*-axis of both hands and wrists, then the corresponding feature vector ***A*** is defined as follows:

$$A=\left(\begin{array}{cccc} \Phi^{\left[hand,x\right]} & 0 & \ldots & 0\\ 0 & \Phi^{\left[hand,y\right]} & \ldots & 0\\ \vdots & \vdots & \ddots & \vdots \\ 0 & 0 & \ldots & \Phi^{\left[wrist,z\right]} \end{array}\right)\in R^{\left(T\cdot D\right)\times \left(M\cdot D\right)}.$$ (5)

With a multi-dimensional formulation, it is possible to learn the correlation between each dimension, which enables inferring the measurement of one dimension given the observations from other dimensions. The optimal weights $W^{⋆}\in R^{\left(M\cdot D\right)\times n}$ can be computed as follows:

$$W^{⋆}={\left(A^{T}A+\lambda I\right)}^{-1}A^{T}\tau .$$ (6)

### 3.4 Modelling a Distribution over a Set of Trajectories

Given a set of trajectories ***τ***, we can compute the parameter posterior *p*(***W***) with the knowledge of *W* ^⋆^ of each single trajectory. We assume $p\left(W\right)=N\left(W|\mu_{w},\Sigma_{w}\right)$, where *μ*_*w*_ and Σ_*w*_ are computed by collapsing the dimension of *n*. A distribution over a set of trajectories *p*(***τ***) can be computed via the marginal over the joint distribution of *p*(***τ***, ***W***), i.e.,

$$\begin{array}{cc} p\left(\tau \right) & =\int p\left(\tau ,W\right)dW\\ & =\int p\left(\tau |W\right)p\left(W\right)dW\\ & =\int N\left(\tau |\Phi W,\Sigma_{y}\right)N\left(W|\mu_{w},\Sigma_{w}\right)dW\\ & =N\left(\tau |\Phi \mu_{w},\Phi \Sigma_{w}\Phi^{T}+\Sigma_{y}\right). \end{array}$$ (7)

With that, we show a probabilistic reconstruction of the demonstrated trajectory set.

### 3.5 Measures for Computing Motion Similarity

One goal of this paper is to quantify the effect of three *non-invasive brain stimulation* approaches tDCS, tACS and tRNS on the human motions. For that, metrics are required to describe the distance between two sets of trajectories, i.e., *p*(***τ*** ^[*A*]^) and *p*(***τ*** ^[*B*]^). According to Stark et al. (2017), *Kullback–Leibler divergence* is the best option for point to point motions, shown as below:

$$D_{KL}\left(P\|Q\right)=\underset{x\in X}{\sum }P\left(x\right)\log\left(\frac{P\left(x\right)}{Q\left(x\right)}\right),$$ (8)

where *P*(*x*) and *Q*(*x*) are two probability distributions defined on the same probability space X. In the case where *P*(*x*) and *Q*(*x*) are equivalent, *D*_*KL*_(*P*‖*Q*) = 0, and KL-divergence increases monotonically with the discrepancy between two probability distributions, namely $D_{KL}\in \left[0,+\infty \right)$.

KL-divergence can effectively measure the distance between two probability distributions, i.e., how much information is lost when approximating one distribution with another. It is broadly used in the field of Machine Learning, e.g., variational autoencoder (Kingma and Welling, 2013) and cross-entropy loss in multi-class classification tasks (Grandini et al., 2020). In deep reinforcement learning, KL-divergence is posed as soft constraints to avoid policy collapse (Schulman et al., 2017; Song et al., 2019). Furthermore, KL-divergence is also used in incipient fault detection (Delpha et al., 2017; Chen et al., 2018).

Note the KL-divergence is asymmetric as *D*_*KL*_(*P*‖*Q*) ≠ *D*_*KL*_(*Q*‖*P*), which is an undesired property for a distance metric. Therefore, to enforce symmetricity, we simply used a trick as in Johnson and Sinanovic (2003), defined as symmetric Kullback-Leibler divergence *D*_*KLS*_. The formulae shown below:

$$\begin{array}{cc} D_{KLS}\left(P\|Q\right) & =D_{KLS}\left(Q\|P\right)\\ & =\frac{1}{2}\left(D_{KL}\left(P\|Q\right)+D_{KL}\left(Q\|P\right)\right), \end{array}$$ (9)

The KL-divergence between two Gaussian distributions is also computationally efficient. Assume $P\left(x\right)=N\left(\mu_{1},\sigma_{1}\right)$ and $Q\left(x\right)=N\left(\mu_{2},\sigma_{2}\right)$, we further have:

$$\begin{array}{cc} D_{KLS}\left(P\|Q\right) & =\frac{1}{2}\left(D_{KL}\left(P\|Q\right)+D_{KL}\left(Q\|P\right)\right)\\ & =\frac{1}{4}\left[\log\left(\frac{\sigma_{2}^{2}}{\sigma_{1}^{2}}\right)+\frac{\sigma_{1}^{2}}{\sigma_{2}^{2}}+\frac{{\left(\mu_{1}-\mu_{2}\right)}^{2}}{\sigma_{2}^{2}}-1\right]\\ & +\frac{1}{4}\left[\log\left(\frac{\sigma_{1}^{2}}{\sigma_{2}^{2}}\right)+\frac{\sigma_{2}^{2}}{\sigma_{1}^{2}}+\frac{{\left(\mu_{2}-\mu_{1}\right)}^{2}}{\sigma_{1}^{2}}-1\right]\\ & =\frac{1}{4}\left[\frac{\sigma_{1}^{2}}{\sigma_{2}^{2}}+\frac{\sigma_{2}^{2}}{\sigma_{1}^{2}}+{\left(\mu_{1}-\mu_{2}\right)}^{2}\left(\frac{1}{\sigma_{1}^{2}}+\frac{1}{\sigma_{2}^{2}}\right)-2\right]. \end{array}$$ (10)

**FIGURE 2:**
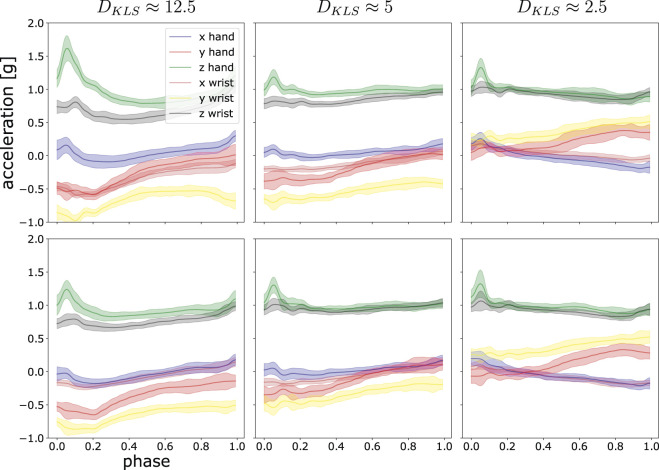
This illustrations shows three examples of the divergent value of *D*_*KLS*_ and the degree of difference in trajectory space. The post-processed acceleration profiles are fitted by ProMPs, with the shaded area being 1*σ* confidence level. In each panel, six motion trajectories of hand and wrist in *x*, *y* and *z*-axis are shown. The top rows are compared with the lower rows to compute *D*_*KLS*_. From left to right, the distance measure *D*_*KLS*_ between the two sets is decreasing.

In our case, we recorded *x*, *y* and *z*-axis for both hand and wrist, altogether six trajectories for one experiment. The symmetric KL-divergence was computed as the averaged *D*_*KLS*_ over six axes. Figure 2 presents a sketch of the correspondence of numerical KL-divergence value to its probability distribution discrepancy. In the case of (coupled) trajectories, we defined the divergent value of two sets of trajectories ***τ***_***1***_ and ***τ***_***2***_ as the mean value by averaging the number of the discrete time points in *phase*, shown as follows:

$$D_{KLS}\left(\tau_{1}\|\tau_{2}\right)=\frac{1}{T}\overset{T}{\underset{t=0}{\sum }}D_{KLS}\left(y_{t}^{1}\|y_{t}^{2}\right),$$ (11)

where $y_{t}^{1},y_{t}^{2}\in R^{6\times n}$ refer to two sets of 6-dimensional measurements at the discrete time point *t* in our case, with each each set containing *n* measurements.

## 4 Experiment Design

In this section, we present the detailed description on experimental design to measure the effect of tACS, tDCS and tRNS on human motions. In addition, we introduce the complete workflow on data post processing.

### 4.1 Sensors

**FIGURE 3:**
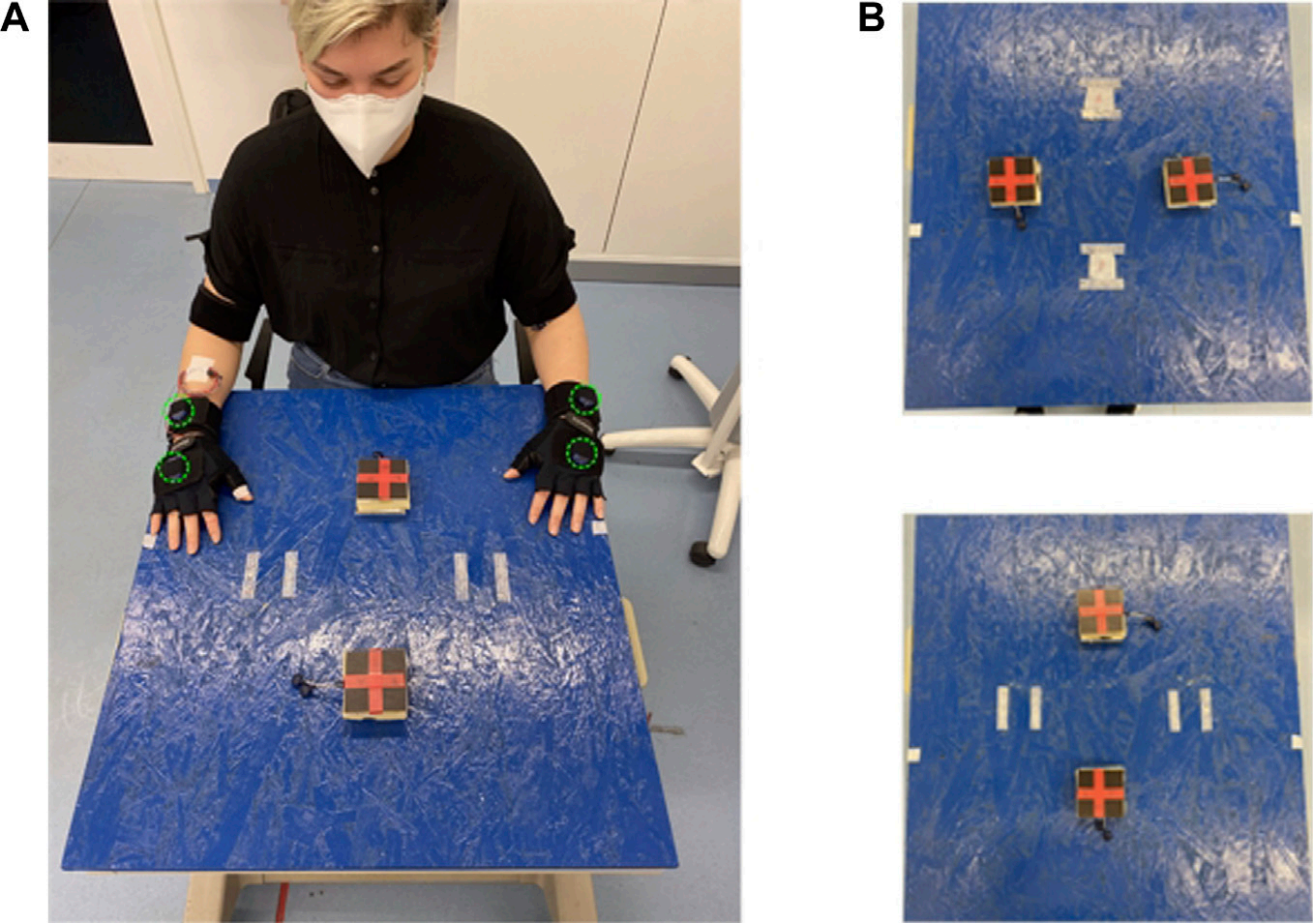
**(A)** Illustration of sensor montage and experiment environments, altogether four IMUs are mounted on subject’s wrists and hands on both sides, marked in green. **(B)** Location of touching pads for different movement direction. **Upper:** left-right tapping direction, **Lower:** posterior-anterior tapping direction. The touch pads provide binary signals which are used to segment the motion signals into individual patters for training the ProMPs, see Section 4.5.

We captured all participants’ motion trajectories by IMUs, i.e., Myon aktos-t sensors. Each aktos-t transmitter includes three-axial sensors for accelerometer, gyroscope and magnetometer. The transmitters are combined with the aktos EMG system for up to 32 channels. We set the sampling frequency of accelerometer, gyroscope and magnetometer to be 2000 *Hz*. We also placed two accelerometers as pressure sensors under the touching pad to split the 1 min recording into movement segments from one pad to the other and vice versa. We mounted totally four sensors respectively on both hands and wrists to capture the arm motion. The recorded sensor profiles will be post-processed for motion difference analysis. The detailed configuration on how sensors are placed is shown in Figure 3A.

### 4.2 Experimental Tasks

In our design, we chose a finger-tapping motion for examining the motion difference under different tCS approaches. In the finger-tapping motion, the subject is asked to perform tapping between two pads with fixed location repetitively. (Benussi et al., 2017; Orrù et al., 2020) also design similar tasks for investigating the effect of tCS variants on coordination. The benefit of a finger-tapping experiment lies in its simplicity for repetitive demonstrations, which is easy for participants to repeat multiple times. Moreover, the degree of freedom of the finger-tapping motions is low and therefore the motion difference can be mostly counted as the effects of tCS approaches. Thirdly, the repetitive motion is also beneficial to ProMPs from a probabilistic perspective, it is more accurate to characterize a probability distribution given more data samples (demonstrations). In total, we defined eight different tapping patterns, (we also call each pattern as unit experiment for the rest of the paper) for each stimuli approach so that the effect of stimuli can be observed in a comprehensive manner. Each experimental setting is shown in Supplementary Table S2. Among them, we distinguished different conditions of tapping with favorable hand, different tapping directions and tapping speed. Below we will discuss the settings for these three categories in detail.

Motion Direction: We set two motion directions, respectively tapping in left-right direction and in anterior-posterior direction, to get the possibility to differentiate between side-dependent and side-independent motions. Figure 3B shows the detailed setting, where the tapping pads are 30 *cm* away from each other.

Motion Speed: We distinguished two movement patterns, i.e., rhythmic and rapid movements patterns. For rhythmic movements, the subject first followed the beep signal corresponding to 1.5 *Hz* to tap for 15 s. The beep signal then vanished and the subject was instructed to go on with the same rhythm for an additional 30 tapping cycles. The total recording time including the beep signal endures 1 min. In the rapid motion case, the subject was instructed to perform tapping as fast as possible for 30 tapping cycles, which lasted from 15 to 30 s.

Motion with favorable hands: We also asked all the subjects to perform the experiments with both hands. Thus, we could also examine to what extent the stimulation approach could impact on favorable/non-favorable hands.

### 4.3 Experimental Protocol

Each participant got measured on 4 days in total. Each of the 4 days was set with 1 week apart from the others to avoid carry-over effects. On each day, the participant received only one type of stimulation, i.e., tACS, tRNS or tDCS, which took up 3 days. We also included a Sham stimulation, which only mimicked the sensory sensation of real tDCS but did not cause any plasticity effects, as it included only 30 s of real stimulation. The stimulation approach was kept unknown from the participant to allow a fair analysis. The recordings of Sham allow us to distinguish whether the difference in motion pattern arises from the neuronal plasticity, i.e., the long lasting changes in the signal transmission induced by the electric stimulation, or repetition only. The order of the tCS protocols including Sham was randomized.

In order to check the influence of the duration of each stimuli, we first familiarized the participant with the eight experiments before the stimuli was activated, All participants could initially practice all eight experiments for a first trial without any stimulus being active. This phase is denoted by the term *Erst*. Going through the experiments once helps reduce the variance of motion patterns for the coming experiments by warming up the participants, as the experiment subject can show different motion patterns, not caused by stimuli but due to the unaccustomedness to the experiment.

Around 45 min after *Erst*, we assume the participants were already familiar with the on-going experiments and also reach a stable state for further experiments, the participants woulf perform all the experiments again (without stimuli), we name the second run *Prae*, which serves as the non-stimulated baseline. After *Prae*, the participant accepted the stimuli and we periodically recorded the motion data around every 35 min until the maximal duration of 115 min are reached. Altogether, we have three recordings after stimuli, namely *post* 1, *post* 2 and *post* 3. Supplementary Table S3 elaborates the procedure.

### 4.4 Participant Information

In this current work, we included an initial study of 10 healthy subjects, all are right-handed. Among them, eight subjects are females. The mean age is 23 years with a range of 20–31 years. All subjects are without reported somatic or psychiatric diseases. The order of each participant’s experiments is shuffled and shown in Supplementary Table S3. The overall number of experiments is 10 ⋅ 4 ⋅ 5 ⋅ 8 = 1,600.

### 4.5 Data Post-Processing

**FIGURE 4:**
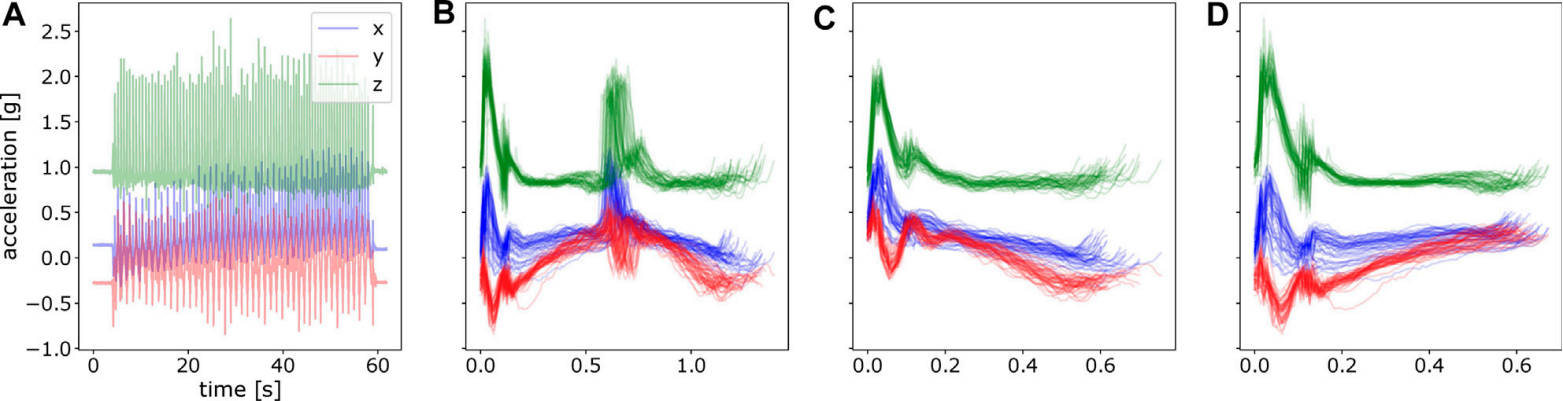
An illustration of raw acceleration profile and segmented data. **(A)** Raw hand acceleration profile of a random subject in one experiment. **(B)** Segmented data for a complete movement from the touching pad 1 to the pad 2 and again to pad 1, concatenating both inward movements and outward movements. **(C)** Segmented motion from the pad 1 to the pad 2, namely, inward movements. **(D)** Segmented motion from the pad 2 to the pad 1, outward movements.

Before applying ProMPs to analyze the motion difference, we first post-processed the data. This includes i) data segmentation ii) alignment and normalization of data over time, i.e., mapping time to the *movement phase* as mentioned in Section 3.2. In data segmentation, we segmented the complete raw trajectory into two sets of point-to-point trajectories, namely inward movements and outward movements respectively. Inward movements refer to the direction where the angle between upper arm and forearm decreases with the motion, vice versa for outward movements. As an illustration, an inward movement for left arm is from left pad to right pad and upper pad to lower pad in Figure 3B assuming the subject’s location is to the lower part of the table. By doing this, we decomposed the motion into stroke-based motions. In these stroke-based motions, we could fit our ProMP model and further compare the difference of non-stimulated patterns against stimulated ones. The segmentation was performed given the statistics of the accelerometer under the touching pad and is shown in Figure 4.

**FIGURE 5:**
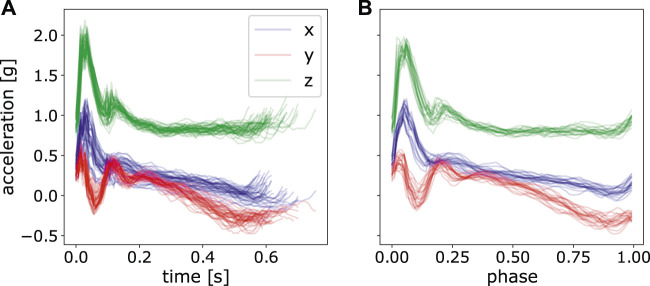
Perform time normalization over all the segmented acceleration data with various time length. **(A)** only segmented data, but not normalized, **(B)** segmented and normalized data of the last 20 strokes.

After segmentation, we could see each segment with various time durations. Since **Φ** is the pre-computed matrix used in Eq. 4, the *phase* of each segmented trajectory must be of the same resolution. And one simple way to achieve that is to perform time alignment and normalization on each segmented piece and then convert the time *t* to the phase *z*_*t*_ as in Eq. 2. It is shown in Figure 5. For fitting model parameters of ProMPs, we took 20 segments from the last twenty-first stroke to the last second stroke. The last stroke was excluded for fitting to avoid subjects’ unintended movement when hearing the stop signal. By this, we have totally 10 ⋅ 5 ⋅ 8 ⋅ 20 = 8,000 strokes of movements for each stimulation approach.

### 4.6 Measuring the Effect of Stimulation Methods

In this subsection, we introduce how we characterized the finger-tapping motions using ProMPs and the motion difference measure *D*_*KLS*_ as defined in Section 3 to measure to what extent and duration of each stimulation approach on motion patterns. We first fit the ProMPs on the segmented and time-aligned data for each of 1,600 experiments, i.e., 1,600 sets of trajectories, as described in Section 4.2. The fitted result showed a probability distribution of the model parameters ***W*** via ***μ***_***w***_ and **Σ**_***w***_. We then mapped the feature ***W*** back to the trajectory space as shown in Section 3.4 so that a probabilistic characterization of the post-processed trajectories ***τ*** was available. We denote such reconstructed trajectories from ProMPs as ***τ′***. To measure the duration of each stimulation’s effect, we computed the mean *symmetric KL-divergence* of *prae* against *post 1*, *post 2* ,*post 3* over all experimental subjects and eight experimental settings in the space of ***τ′***, namely:

$$\begin{array}{cc} & D_{KLS}\left({\tau \prime }_{\left[o=prae\right],p,q}\|{\tau \prime }_{\left[o=post\;1\right],p,q}\right),\\ & D_{KLS}\left({\tau \prime }_{\left[o=prae\right],p,q}\|{\tau \prime }_{\left[o=post\;2\right],p,q}\right),\\ & D_{KLS}\left({\tau \prime }_{\left[o=prae\right],p,q}\|{\tau \prime }_{\left[o=post\;3\right],p,q}\right), \end{array}$$ (12)

for each *p* and *q* on all available data for inward movements and outward movements respectively, where *o* refers to the four experimental phases of *prae*, *post 1*, *post 2* and *post 3*, and *p* denotes the index of different unit experiments. The index *q* stands for the number of participants. We excluded *Erst* for statistical analysis as the purpose of *Erst* is to familiarize the subject with experiment and the subjects are likely to perform exploratory behaviors for each unit experiment.

## 5 Results

In this section, we explain in detail how we computed the trajectory difference using *probabilistic movement primitives* (ProMPs) to reveal the effect of different *transcranial current stimulation* methods on human arm motion. We present comprehensive results using all of the available data to show that ProMPs together with the *symmetric KL-divergence* can be used for characterizing trajectory differences in brain stimulation studies. We proceed in a progressive manner by answering the following questions: i) Which sensor profile is best suited to analyze stimulation methods? (acceleration, velocity or displacement profile) ii) What is the reconstruction error using ProMPs? iii) Can ProMPs be used to detect outliers? iv) How are the effects of tRNS, tDCS and tACS over the finger-tapping motion? v) Can time-specific differences be detected on a millisecond time scale?

### 5.1 Which Sensor Profile is Best Suited to Analyze Stimulation Methods?

**FIGURE 6:**
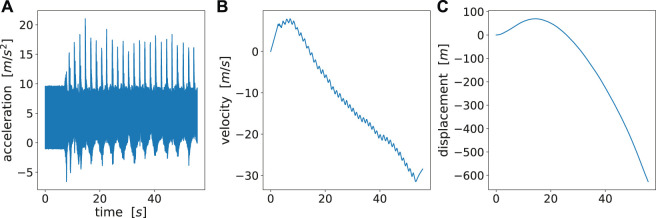
**(A)** Raw acceleration data, **(B)** reconstructed velocity profile, **(C)** reconstructed acceleration profile. There is a large drift for velocity and displacement as no periodic patterns can be observed. A reasonable reconstruction should at least show periodic patterns as acceleration profile.

We retrieved the raw magnetometer, gyroscope and accelerometer readings from the inertial measurement units (IMUs) attached to both wrists and hands. There are three options where we could fit ProMPs, either on displacement profile *s*(*t*) or velocity profile *v*(*t*) or acceleration profile *a*(*t*). We also derived the velocity and displacement profiles using Attitude Heading Reference System Filter (Ahrs Filter) (Roetenberg et al., 2005) as shown in Figure 6. However, it could be observed that the reconstructed *v*(*t*) and *s*(*t*) were largely distorted due to the (double) integration over time. The distortion of the reconstructed signal arose from the inaccurate recordings from in orientation or acceleration, where the error was accumulated and amplified after performing double integration over time (Kok et al., 2017).

Fortunately, the acceleration profile does not require any time integration, hence bears the highest accuracy among these three options. Besides, it well explains the motion difference among different trajectories. The motion difference could still be shown in the acceleration profiles during the motion phase. For these reasons, we directly fit ProMPs on raw acceleration data. One pitfall of directly using raw acceleration profiles is that the raw acceleration can vary if the initial poses are different. To avoid that we precisely instructed the subjects on how to place their hands at the start of each experiment.

### 5.2 What is the Reconstruction Error Using ProMPs?

To show the quality of the fitted model of ProMPs on the dataset, we computed the *D*_*KLS*_ between the reconstructed trajectories from ProMPs and the post-processed ones on different hyper-parameter settings. In Table 1, we list the reconstruction error defined as:

$$L_{rec}=\frac{1}{\left|o\right|\cdot \left|p\right|\cdot \left|q\right|}\underset{o,p,q}{\sum }D_{KLS}\left(\tau_{o,p,q}\|{\tau \prime }_{o,p,q}\right),$$ (13)

where *t* refers to the number of discrete time points after time normalization, i.e., phase *z*, and ***τ*** and ***τ′*** are respectively the set of post-processed trajectories and the set of reconstructed trajectories fitted by ProMPs as shown in Section 3.4. Additional hyper-parameters of ProMPs are the centers *c*_*i*_ and the bandwidth *h*_*i*_ in Eq. 2 respectively. In our case, we set *h*_*i*_ as a function of the number of basis functions *M*, so that the tunable hyper-parameter is merely *i*. The setting of bandwidth of each basis function goes as $h=0.2\cdot {\left(c_{i+1}-c_{i}\right)}^{2}$ and the centers *c* are uniformly distributed between the phase of [0, 1]. It can be seen that the reconstruction loss decreases with the increasing number of basis functions. Although even better reconstruction loss can be achieved by increasing *M*, it risks overfitting from a machine learning perspective. We discuss this point in Section 6. From a machine learning perspective, the raw data is usually projected into a feature space of smaller dimension than raw data.

**TABLE 1:** Reconstruction loss with respect to number of basis functions in ProMPs. The reconstruction loss is computed using *D*_*KLS*_(***τ***‖***τ′***). Here we show the mean and standard deviation on the reconstruction loss from three randomly chosen participants, corresponding to 3 ⋅ 8 ⋅ 4 ⋅ 4 = 384 sets of trajectories. Each set of trajectories consists of 20 single demonstrations.

Number of basis functions *M*	Reconstruction loss *L*_*rec*_
5	0.207 ± 0.049
10	0.073 ± 0.019
15	0.034 ± 0.011
20	0.016 ± 0.005

In the remaining experiments, we set the hyper-parameters of ProMPs as: *n* = 20, *h* = 0.2 ⋅ (20–1)^−2^, and the regularizer term *λ* = 1*e*-6.

**FIGURE 7:**
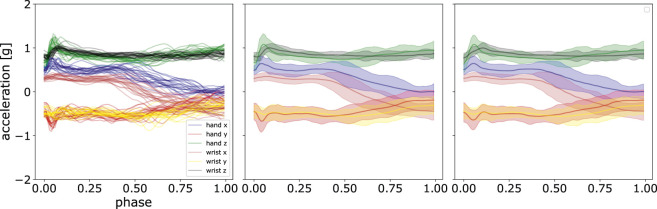
Comparison of the mean and standard deviation of the post-processed acceleration profiles and the reconstructed acceleration profiles using ProMPs. The left panel shows the data after post-processing, the middle panel displays the mean and standard deviation of that data, and in the right panel, the reconstruction using ProMPs is illustrated. The shaded area corresponds to 2*σ* confidence interval.

An illustration using these parameters is shown in Figure 7. It can be observed that ProMPs accurately model the recorded data. One can also see that the trajectories can be effectively modelled using uni-modal Gaussian distribution at each discrete time point.

### 5.3 Can ProMPs be Used to Detect Outliers?

**FIGURE 8:**
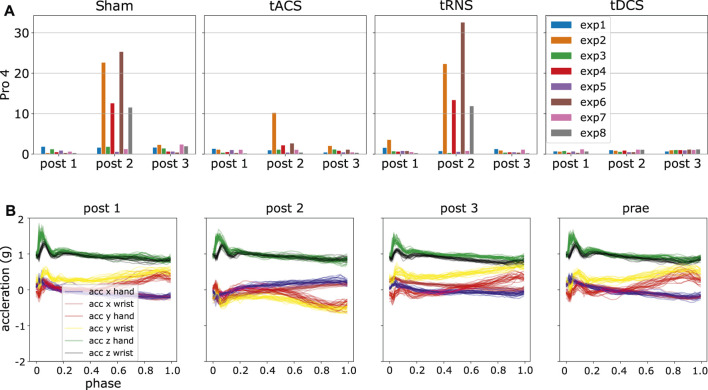
Using ProMPs with symmetric KL-divergence helps detect potential data anomalies. **(A)** Potential anomalies in high KL-divergence case, i.e., high *D*_*KLS*_ case **(B)** Flipping of the blue, red, brown and yellow curves, i.e., *x*, *y*-axis recordings in hand and wrist in *post 2*.

We generated the *D*_*KLS*_ in Eq. 9 for each participant and experiment according to the steps in Section 4.6. By analyzing the resulting statistics, we detected some potential anomalies. An initial observation is that *D*_*KLS*_ is small among most of the experiments and subjects. However, there are peaked values in few cases, suspected as outliers. For instance, the irregular patterns (high divergent value) in the experiment 2, 4, 6, 8 in the day of Sham and tRNS for participant four are present, as shown in Figure 8. A closer look into the post-processed data revealed that the *x*, *y*-axis recordings for both hand and wrist were flipped, a sign indicating the hand and wrist IMUs were misplaced. We also verified this hypothesis by looking into the recorded video and confirm the sensor misplacement. Similar problems of sensor misplacements were also observed in participant 11 in experiment 2, 4, 6, 8 in *post* 1, tDCS.

**FIGURE 9:**
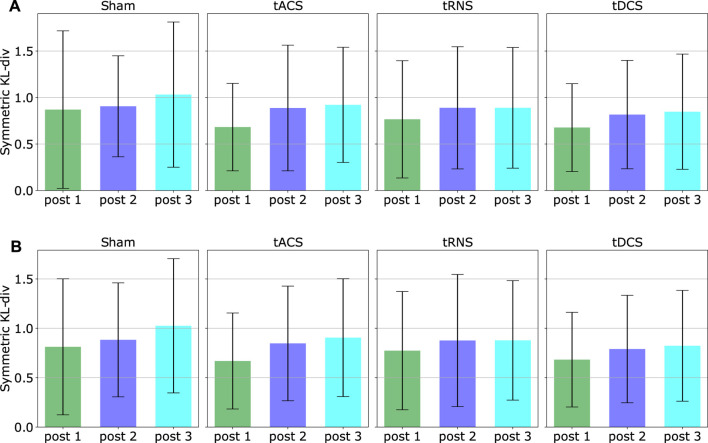
An illustration of ProMPs with *D*_*KLS*_ detecting data outliers. **(A)** High *D*_*KLS*_ case in participant 19, tDCS, the sixth experiment, brown bar shows remarkably higher value than others, indicating a potential outlier. **(B)** Post-processed data for the experiment 6 in tDCS. The yellow curve accounts dominantly for the high divergence, interpreted as a different motion behavior in *Prae* from other three.

Besides the man-made mistakes, there are also other high divergent cases, e.g., shown in Figure 9. To automatically detect the data anomalies, we used the 3*σ* rule, which is equivalent to a confidence interval of [0.15*%*, 99.85*%*]. Samples are regarded as outliers if *D*_*KLS*_ in Eq. 11 is larger than *μ*_*D*_ + 3*σ*_*D*_, i.e., higher than 99.85*%* confidence level in either inward or outward movements. The analysis of high *D*_*KLS*_ values is presented in Section 6.5. Samples with *D*_*KLS*_ values below 0.15*%* confidence level are valid and were not excluded from the evaluations. Here *μ*_*D*_ refers to the mean value of *D*_*KLS*_ averaged over all non-corrupted experiments and participants, and *σ*_*D*_ is the corresponding standard deviation. We removed such outliers for the further statistical analysis. By this we show that the ProMPs with *symmetric KL-divergence* can effectively detect anomalies, which is much more efficient than manual investigation of 1,600 sets of trajectories.

### 5.4 How are the Effects of tRNS, tDCS and tACS Over the Finger-Tapping Motion?

In this section, we present the results on the motion difference under different *Transcranial Current Stimulation* methods. The difference is quantified by *D*_*KLS*_ and ProMPs following the procedure in Section 4.6 and Section 5.3. Supplementary Figure S1 shows the statistics of *D*_*KLS*_ of each participant and experiment, excluding the outliers and corrupted data.

**FIGURE 10:**
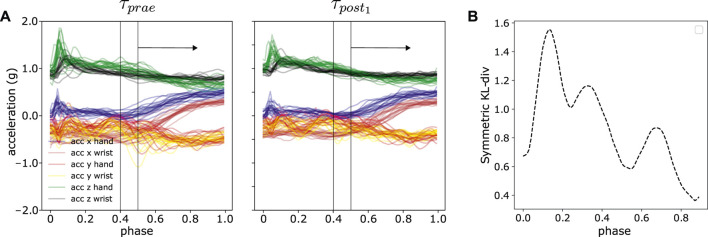
This Figure shows the *D*_*KLS*_ value of experiment subject 2 for each stimulation approach, each experiment configuration and each post-stimulation phase. The *D*_*KLS*_ are computed from the outward movements. The x-axis denotes the comparison between post-stimulation phase *Post* and non-stimulated phase *Prae*. The statistics already excludes the outliers via the 3*σ* rule mentioned in Section 5.3. As the *D*_*KLS*_ is quite low, no significant effect of tACS, tRNS and tDCS over the hand motion can be observed in this subject.

We illustrate the statistics from one random participant in Figure 10. By looking at the figure, the value of *D*_*KLS*_ in most cases are around 1. According to Figure 2, the trajectory difference is insignificant in the case of *D*_*KLS*_⩽2.5. In this participant, the divergent value implies no pronounced discrepancy is observed in each type of stimuli or different post-stimulation phases. In addition, we checked whether the effect of stimuli varies across the post-stimulation phases of 45, 90 and 135 min after the stimulation is activated. In this participant, we did not observe any trends among different post-stimulation phases. Furthermore, the divergent values in Sham are similar to those in tACS, tDCS and tRNS for all eight experiments and post-stimulation phases. This indicates different stimulation approaches do not exert notable effects on movements on this experimental subject. The divergent value of 1 mainly comes from the stochasticity of the participant’s movement, i.e., the participant cannot reproduce the exact same movement in different trials.

**FIGURE 11:**
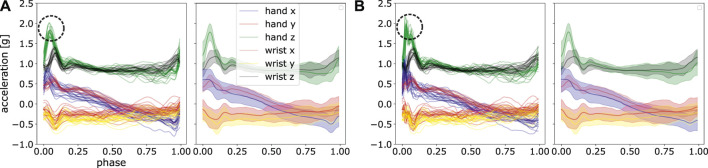
The figure illustrates an overall effect different stimulation type and post-stimulation phases. The means and standard deviations of each *D*_*KLS*_ values are computed by averaging all 10 subjects and eight experiment configurations, respectively using the trajectories of **(A)** inward movements **(B)** outward movements. The outliers have been excluded.

We computed the mean and the standard deviation of the *D*_*KLS*_ value in that figure over all participants and all eight experiment configurations based on both inward and outward movements respectively. The result is shown in Figure 11. With the given 10 subjects, it can be seen that the averaged *D*_*KLS*_ values are smaller than or around 1, which shows no remarkable difference in terms of stimulation type or post-stimulation time. The standard deviations of the *D*_*KLS*_ remain similar among different stimulation approaches and inward/outward movements. No specific trends in standard deviation can be observed between post-stimulation phases. We could not observe a statistically significant difference when comparing the baseline approach Sham to three other stimulation approaches. Potential reasons are discussed in Section 6.6.

A comprehensive result on each subject and experiment is further illustrated in Supplementary Figure S1.

### 5.5 Can Time-Specific Differences be Detected on a Millisecond Time Scale?

**FIGURE 12:**
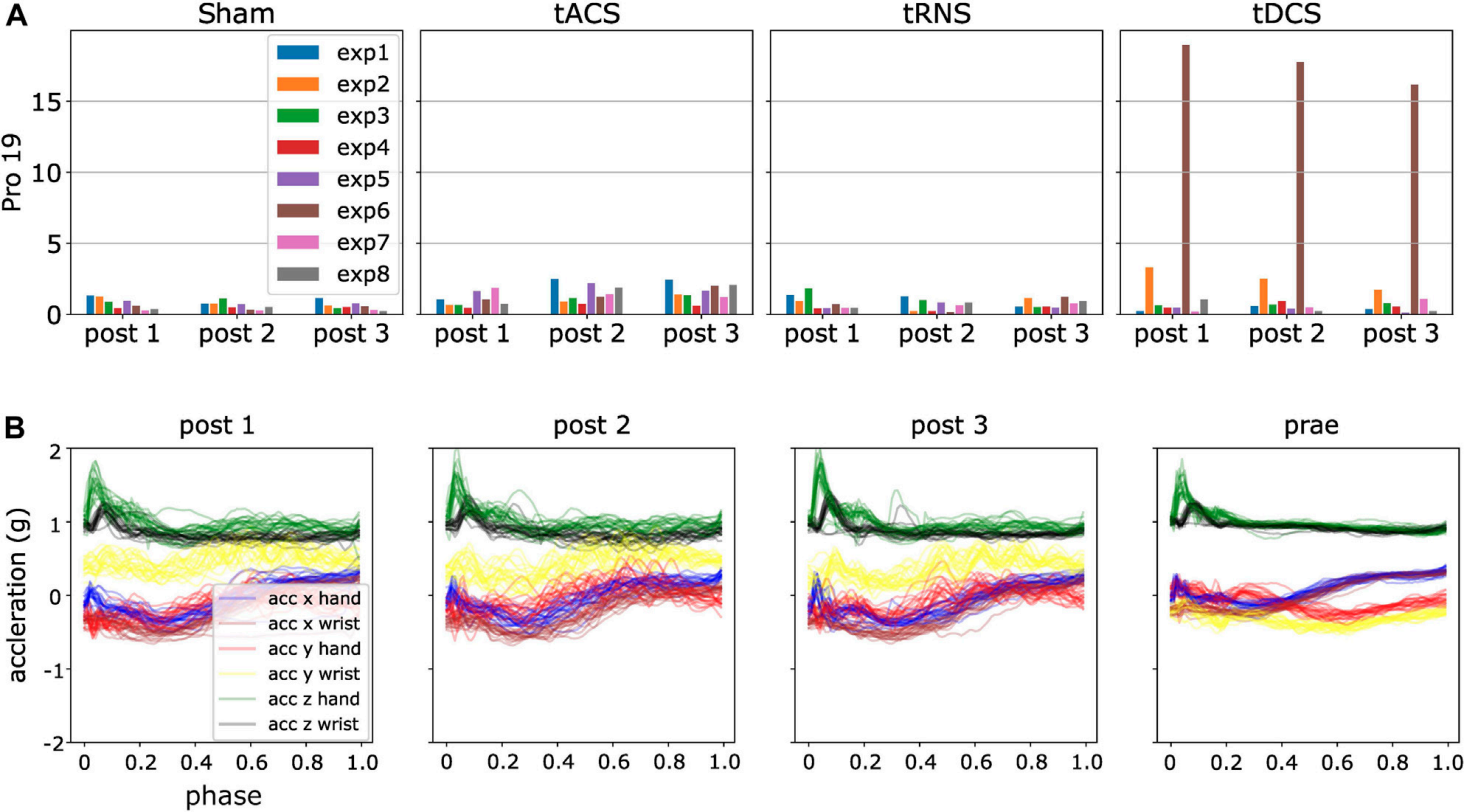
An illustration of sliding window approach to reveal time-specific difference in trajectory space. **(A)** Sliding window with the size of 1/10 of the total normalized time, i.e., *phase* between ***τ***_*prae*_ and ***τ***_*post*__1_ from one random experiment **(B)** The resultant symmetric KL-divergence averaged over all six axes between two experiments in sliding window manner.

The analysis in Section 5.4 presented a compact distance measure between two sets of trajectories by averaging over the discrete timepoints, shown in Eq. 11. In this part, we further examined the per-time-step distances, i.e., which part of the trajectories contribute to the motion difference by using a sliding window approach. The per-time-step distances provide detailed insights on a millisecond time scale. The window size is pre-defined and we computed the *D*_*KLS*_ within the window, slid across the *phase* *z*_*t*_. The resultant curve illustrates the general trends of difference in relation of time. The sliding window approach can extract the most significant difference or features between two sets of trajectories as demonstrated in Figure 12. We provided a comprehensive outlook on the trends of trajectory distances using sliding window approach in Supplementary Figures S2, S3 for each subject and each experiment. In some cases, e.g., subject 7 in experiment 3, 4 and 6, the most peaked difference is within 0–20*%* and 80–100*%* of the *movement phase* in Figure 12B. The first 20*%* phase in our experiments corresponds to contacting the source pad and subsequently lifting the hand, whereas 80–100*%* phase corresponds to dropping the hand or approaching the target pad. On the other hand, some subjects display semi-uniform motion differences across the entire movement phase, e.g., subject 13 in experiment 2. However, there is no specific general patterns among subjects. These interesting observations will be further investigated in future experiments with a larger number of subjects.

## 6 Discussion

In this section, we first discuss the advantages and limitations of our approach ProMPs for modelling human motions and also shed light on the effects of the *non-invasive brain stimulation* methods tACS, tRNS and tDCS on the finger-tapping motions.

### 6.1 Efficiency of ProMPs in Motion Modelling

As an auxiliary analysis tool, ProMPs with *symmetric KL-divergence* provides an efficient way of motion analyses compared to manual investigations. The computational time needed for learning ProMPs and computing the *symmetric KL-divergence* takes less than 10 min for all 1,600 experiments, i.e., 1,600 ⋅ 20 = 32000 single stroke of movements. The duration of 10 min also includes the time spent for the expensive frequent read-write processes, i.e., loading post-processed data and saving the statistics. In contrast, the entire analysis time could endure days for the same amount of data in human case.

### 6.2 ProMPs with Symmetric KL Divergence as a Consistent and Robust Distance Metric

We also show that ProMPs together with *symmetric KL-divergence* constitute a consistent metric in quantifying the difference between sets of trajectories. One direct way is to compare the difference between the post-processed trajectories and fitted ones using ProMPs as shown in Table 1. With a proper choice of the number of basis functions *M*, the mean value of reconstruction loss and its standard deviation over the available data is sufficiently small. This result validates ProMPs as a robust and consistent feature extractor on the collected motion data.

Additionally, we compared the *D*_*KLS*_ value respectively for both inward and outward movements on each experiment as shown in Supplementary Figure S1. We further computed the mean and standard deviation by collapsing all the dimensions, i.e., experiment configurations, the number of experimental subjects, stimulation approaches and post-stimulation phases. The result is shown in Table 2. As a consistent and robust distance metric, they should hold similar outcomes.

**TABLE 2:** A comparison of the averaged *symmetric KL-divergence* on both inward and outward movements over all experiments and participants, the mean and standard deviation are computed by averaging each *D*_*KLS*_ values in Supplementary Figure S1.

	Averaged *D*_*KLS*_
Inward movements	0.874 ± 0.689
Outward movements	0.865 ± 0.695

As can be seen from Supplementary Figure S1 and Table 2, the *symmetric KL-divergence* is highly similar for all inward and outward movements. The similarity is expected as the divergent values *D*_*KLS*_ measured from inward and outward movements should be close to each other. This also verifies the consistency of ProMPs and *D*_*KLS*_ as a measure of difference between trajectories.

Another benefit is the robustness of ProMPs against noise as a feature extractor, which is critical for further analysis of medical methods. In some manually-designed features, the noise extensively affects the feature extraction and consequentially leads to wrong classification or distance metric computation. For instance, in (Bologna et al., 2016), they defined the maximal velocities and others from the complete trajectory as the features, which were highly-sensitive to noise. In our work, the noise mainly arose from three potential aspects: i) IMU sensor noise ii) movement outliers iii) unexpected oscillations or shifts of IMUs during intense movements.

**FIGURE 13:**
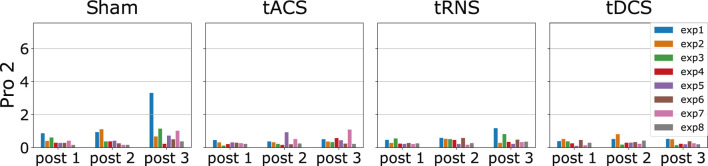
We provide a set of post-processed trajectories with filtering **(A)** and without filtering **(B)**. Frequencies higher than 20 *Hz* are filtered out. A high similarity between fitted results using ProMPs can be observed, especially the automatic filtering effect can be seen from the green curve at its peak value, marked in circle.

In ProMPs, however, the noise is filtered through applying the basis functions. The robustness against noise stems from the shape of basis functions and the number of the basis functions used. High-frequency noise can be interpreted as a non-smooth jerk, which cannot be perfectly fitted using a limited number smooth radial basis functions. Therefore, the noise is automatically left out in the process of model learning with a suitable choice on model hyper-parameter. Note that by having a extremely small bandwidth of the basis functions (see *h* in Equation 2) and a large number of *M*, it is theoretically possible to even fit to noise, i.e., the model can overfit. In ProMPs, high frequency artefacts are typically not pronounced. In contrast, additional step of filtering is always necessary in frequency-domain models. Figure 13 illustrates a visual comparison of non-filtered data and a filtered one, where one can observe a noise-filtering effect on the reconstructed trajectories. Moreover, the reconstructed trajectories using filtered data is highly similar to the ones using non-filtered data, which is desired. Given sufficient demonstrations, ProMPs is also robust against outliers, as the mean and standard deviation will not be shifted greatly for a set of demonstrations.

### 6.3 Generalizability of ProMPs to Other Motion-Modelling Tasks

One clear advantage using ProMPs compared to other manual feature design approaches is its generalizability to out-of-domain tasks only with minimal efforts in hyper-parameter tuning. The center *c*_*i*_ and the bandwidth *h*_*i*_ of each basis function are automatically adapted according to the number of basis functions *M*. The only true open parameter is *M*, which is adapted based on the non-linearity or complexity in the data. Typical values for *M* are in the range of 10–30. The key of ProMPs’ generalizability to other task lies in the extraction of key features representing the trajectory shape, which allows to quantify similarities or to visualize clusters. In Rueckert et al. (2015), the model was extended to also learn latent robot control parameters in a two-dimensional space. Such approaches can be also used for visualizing and analyzing complex human motion data.

### 6.4 Limitations Using ProMPs

In this part, we also discuss the limitations of ProMPs. First, a proper selection of hyper-parameter is necessary to achieve desired feature extraction. This is also referred to as the general challenge in machine learning, the mean-variance tradeoff. As can be seen in Table 1, an insufficient number of basis function *M* can cause a high bias, whereas high model complexity risks modelling undesired motion features, i.e., noise in extreme cases. Another point is that at least two trajectories must be present for fitting ProMPs to enable mean-variance scheme from a theoretical perspective. In theory, it is always better to have as plentiful demonstrations as possible. From a statistical view, more samples drawn from an unknown probability distribution leads to better approximation of the ground-truth distribution. This in turn poses the requirement that the tasks should be performed repetitively. However, in practice typically 10–30 repetitions are sufficient.

It is also noteworthy that ProMPs require the time-alignment or normalization to make each segmented data have the same length. By performing time alignment and normalization, the model diminishes the difference between trajectories by ignoring the actual duration of each segments.

### 6.5 Potential Causes of Outliers

As mentioned in Section 5.3, before applying the 3*σ* rule to exclude outliers, we indeed saw a few individual cases of high *D*_*KLS*_ values. For instance, we observed a peaked *D*_*KLS*_ value in the sixth experiment in tDCS from participant 19 as shown in Figure 13. It shows that the post-processed trajectories in *Prae* display a different pattern from *Post 1-3*, whereas the trajectories in different post-stimulation phases are similar. Nonetheless, the difference is only noted in the sixth experiment in this participant, whereas the divergent values for all other experiments are similar to each other and well below the one in the sixth experiment. We deemed such cases as outliers, and it was automatically ruled out with 3*σ* criterion.

The potential cause of such high divergent values is that a participant attempted to finish the experiment in a different manner or the participant’s initial pose for each experiment is deviating, despite the fact that each participant was told to start with the same initial pose and same motion behavior for each experiment as much as possible. Some subjects might still be in the process of getting familiar with the experiments so that they performed exploratorily between experiments. In some cases, they forgot the initial pose after the interval of around 45 min between each post-stimulation approach so that the acceleration profiles in local coordinate frames exhibit different patterns.

### 6.6 The Effects of tACS, tDCS and tRNS on Finger-Tapping Motions

In this work, the effects of tACS, tDCS, and tRNS are quantified using ProMPs and *D*_*KLS*_. If the divergent values of the stimulation approaches are different from the baseline approach Sham, the effect of the stimuli on the motion can be indeed inferred. However, the results in Section 5.4 shows that no specific effects can be seen from tACS, tDCS, or tRNS using our approach. Previous works (Grimaldi et al., 2014) mentioned that an effect of the stimulation on the neural excitabilities can be observed. However, the neural excitability is not necessarily reflected in the arm motions. It remains an open research question and our initial results does not display notable effects.

As illustrated in Figure 10, we noticed a marginal increase in the mean divergent values of all stimuli types including Sham with the increasing post-stimulation phases. This marginal increase might be caused by an increasing tiredness or an degradation of attention or free activation of muscles from the subjects, e.g., sleeping sideways or long-time sitting posture and other factors during the experiments. For a total duration of the experiments of 4 h, it could be necessary to add more resting phases which will be investigated in future experiments.

We also included Supplementary Figure S4 to demonstrate the stimulation effects at individual level. It can be seen that all subjects displays *D*_*KLS*_ values lower than 2 for all stimulation types and post-stimulation phases, i.e., no remarkable difference can be observed. We further examined along the dimension of each stimulation type (each column) to check whether there was a general effects or trends. And we did not discover a general pattern for each stimulation type. For instance, subjects 11 and 19 showed a marginal increase in tACS with the increasing post-stimulation phase, whereas subjects eight and nine did not show such trends. An in-depth analysis on individual difference is only possible with more detailed information of each subject and a larger number of subjects, which is a new research question.

However, we do not conclude that tACS, tDCS or tRNS plays no an effect on human motions in this work for several reasons. Firstly, the data collection is still on-going, it would be statistically more significant to have more data. We planned to integrate 30 participants at the end. Another point is that ProMPs cannot capture the effect of the time duration of each motion segments, on which these brain stimulation approaches could take effect. According to some other work (Benussi et al., 2017; Orrù et al., 2020), tCS approaches could also influence the accuracy of tapping, i.e., whether the finger taps the middle point of the touching pad. The subjects could potentially adjust the trajectory with the presence of the plasticity effect, where the fitted acceleration profile can only partially show this adjustment effect. The ideal case to examine this is the montage of additional sensors to detect the touching point.

## 7 Conclusion

In this paper, we proposed *Probabilistic Movement Primitives* (ProMPs), a well-established approach in robot skill learning, for human motion analysis to examine how variants of *Transcranial Current Stimulation* approaches affect human motions in finger-tapping experiments based on IMU recordings. We showed ProMPs together with the *symmetric Kullback-Leibler divergence* constituted a robust distance metric for measuring the difference between sets of motion trajectories. As an auxiliary analysis tool, ProMPs first provide much faster diagnosis and more objectivity than human experts and can even reveal potential experimental mistakes, e.g., sensor misplacement. Compared with other methods that rely on manually-designed features or frequency-domain analysis, ProMPs are robust against noise and extract features of the trajectory shape, which makes it easily extendable to other tasks. In ProMPs, merely a single model hyper-parameter, namely the number of basis functions, needs to be defined or determined. In this paper, we discussed how this parameter can be determined by analyzing the reconstruction error. We also demonstrated and discussed how ProMPs can be used for filtering noise which tremendously reduced the required human effort for data post-processing.

In the current work, we discussed the effect of transcranial random noise stimulation (tRNS), transcranial alternating current stimulation (tACS) and transcranial direct current stimulation (tDCS) on finger-tapping motions using an initial data of 10 participants. In our initial study, our approach did not reveal any significant effects of these stimulation approaches on the tapping movement. In future work, we will further increase the number of subjects to validate our hypotheses through statistically significant results. We will also consider other experiments such as lifting the objects and pick-placing little objects characterized by arm movements (Herzog in preparation, Cerebellar transcranial current stimulation revised – an intraindividual comparison). Additionally, we will also investigate other distance measures listed in Stark et al. (2017) together with other outlier-exclusion schemes in Chen et al. (2018).

## Data Availability

The raw data supporting the conclusions of this article will be made available by the authors, without undue reservation.
