# Supplementary material for: Using Probabilistic Movement Primitives in Analyzing Human Motion Differences Under Transcranial Current Stimulation
Source: Front Robot AI. 2021 Sep 14;8:721890. doi: 10.3389/frobt.2021.721890 (PMC8476753; doi:10.3389/frobt.2021.721890)
Supplement: Supplementary file 1 [file DataSheet1.pdf]

Supplementary Material

In this section, we elaborated the details of the experimental schedule within each day and the randomized order of each participant on accepting the stimulation approaches of tACS, tDCS, tRNS and Sham. We also showed how each unit experiment was configured. In addition, a complete statistics on characterizing the effects of stimulation using D_{KLS} with ProMPs mentioned in Section 3, and the time-specific difference with a sliding window approach on each participant, experiment and post-stimulation phase was illustrated.

Time w.r.t. stimuli	Day 1 Sham	Day 2 tACS	Day 3 tRNS	Day 4 tDCS
-90 min	Erst	Erst	Erst	Erst
-45 min	Prae	Prae	Prae	Prae
35 min	Post 1	Post 1	Post 1	Post 1
75 min	Post 2	Post 2	Post 2	Post 2
115 min	Post 3	Post 3	Post 3	Post 3

Table S1. Illustration of experimental schedule of participant 1, negative value means prior to an event, positive value means post to an event. Note each day, i.e., Day 1, Day 2, Day 3 and Day 4 are separated from each other by one week. We also instructed every participant to stick to the same motion pattern as much as possible to avoid the effect of exploring different motion patterns to finish the experiment, i.e., reduce the trajectory variance not caused by stimuli.

Experiment index	Tapping direction	Rhythmic / Rapid motion	Right / Left hand
1	left-right	rhythmic	right hand
2	left-right	rhythmic	left hand
3	left-right	rapid	right hand
4	left-right	rapid	left hand
5	forward-backward	rhythmic	right hand
6	forward-backward	rhythmic	left hand
7	forward-backward	rapid	right hand
8	forward-backward	rapid	left hand

Table S2. A detailed illustration on the configuration of eight finger-tapping experiments.

Subject index	Day 1	Day 2	Day 3	Day 4
1	Sham	tACS	tRNS	tDCS
2	tACS	Sham	tRNS	tDCS
4	tDCS	Sham	tRNS	tACS
7	tRNS	tACS	tDCS	Sham
8	tDCS	Sham	tACS	tRNS
9	Sham	tDCS	tACS	tRNS
11	tACS	tRNS	tDCS	Sham
13	tDCS	tACS	Sham	tRNS
15	tACS	tRNS	Sham	tDCS
19	tACS	tDCS	Sham	tRNS

Table S3. Shuffled order on stimulation approach. Note there are skips between subject indices as the experiments of the subject with the skipped indices are still on-going.

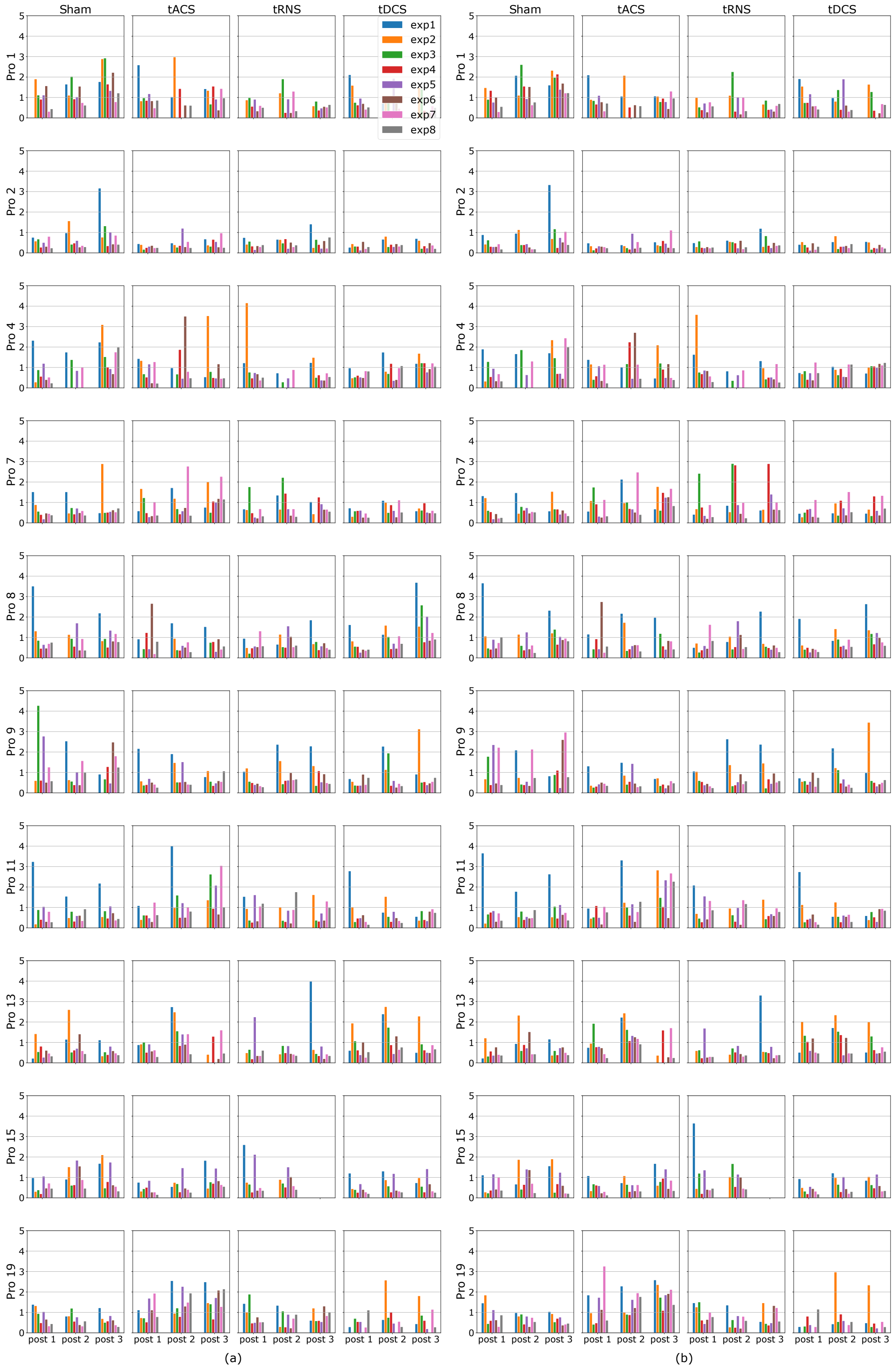

Figure S1: Trajectory difference measured by ProMPs and *symmetric KL-divergence* on 10 participants. The result is demonstrated in terms of each experiment setting, stimulation method and each phase after stimulation. (a) Distance measured on all the segmented inward movements for arm (b) Distance measured on all the segmented outward movements for arm. The missing bars correspond to the filtered outliers.

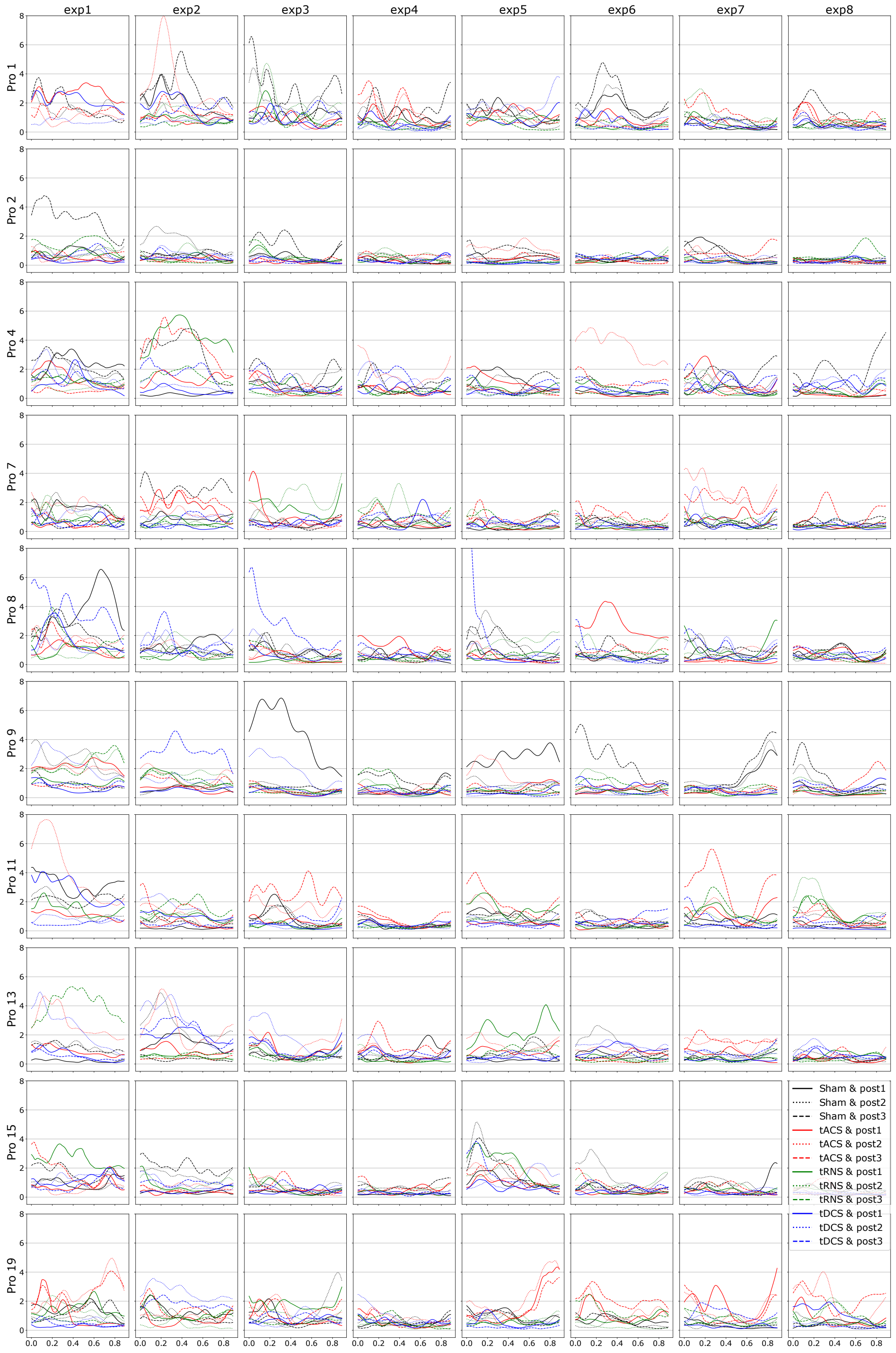

Figure S2: Sliding window for time-specific trajectory difference on all inward arm movements, measured via ProMPs and *symmetric Kl-divergence*. The outliers have been excluded.

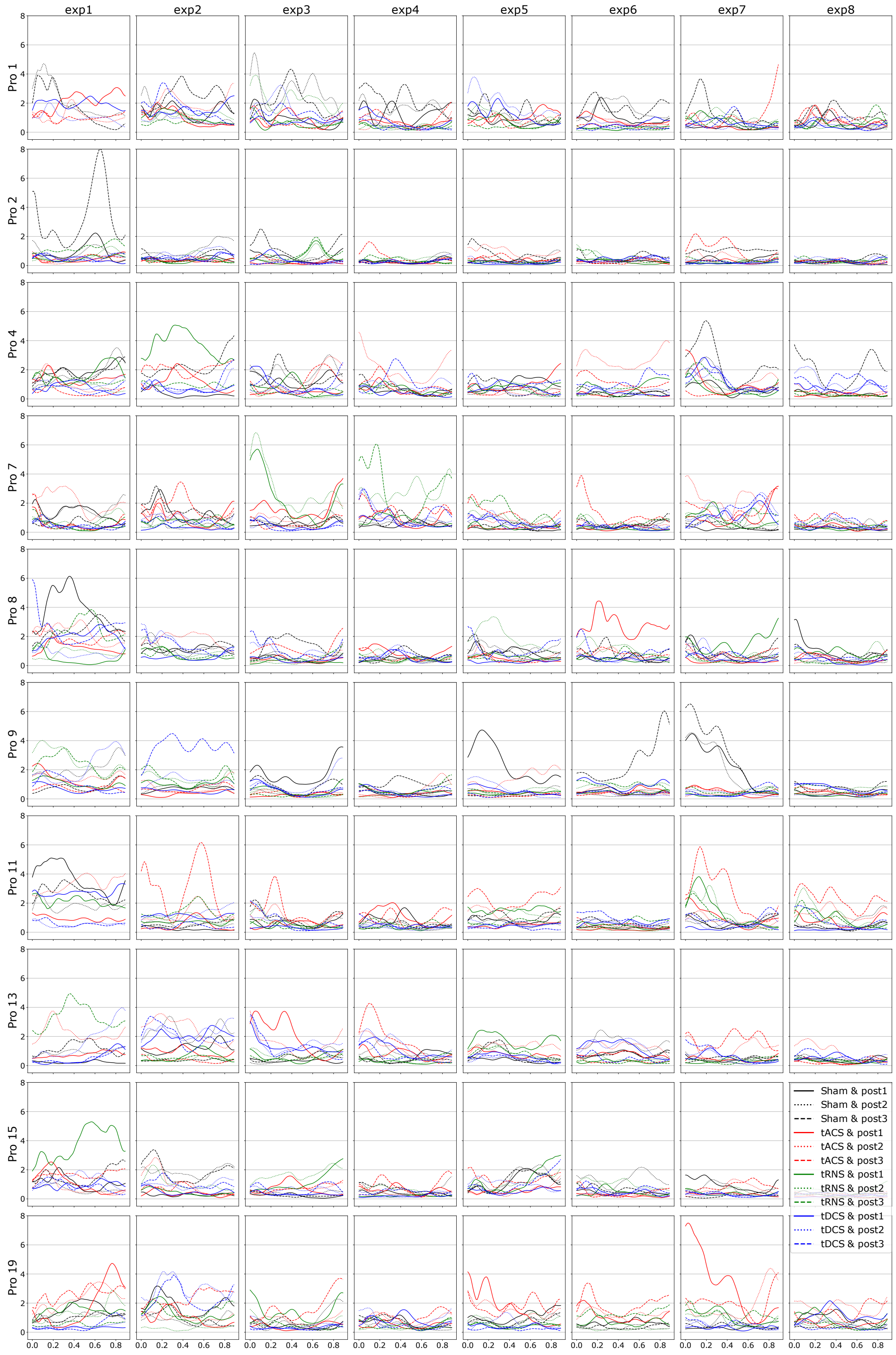

Figure S3: Sliding window for time-specific trajectory difference on all outward arm movements, measured via ProMPs and *symmetric Kl-divergence*. The outliers have been excluded.

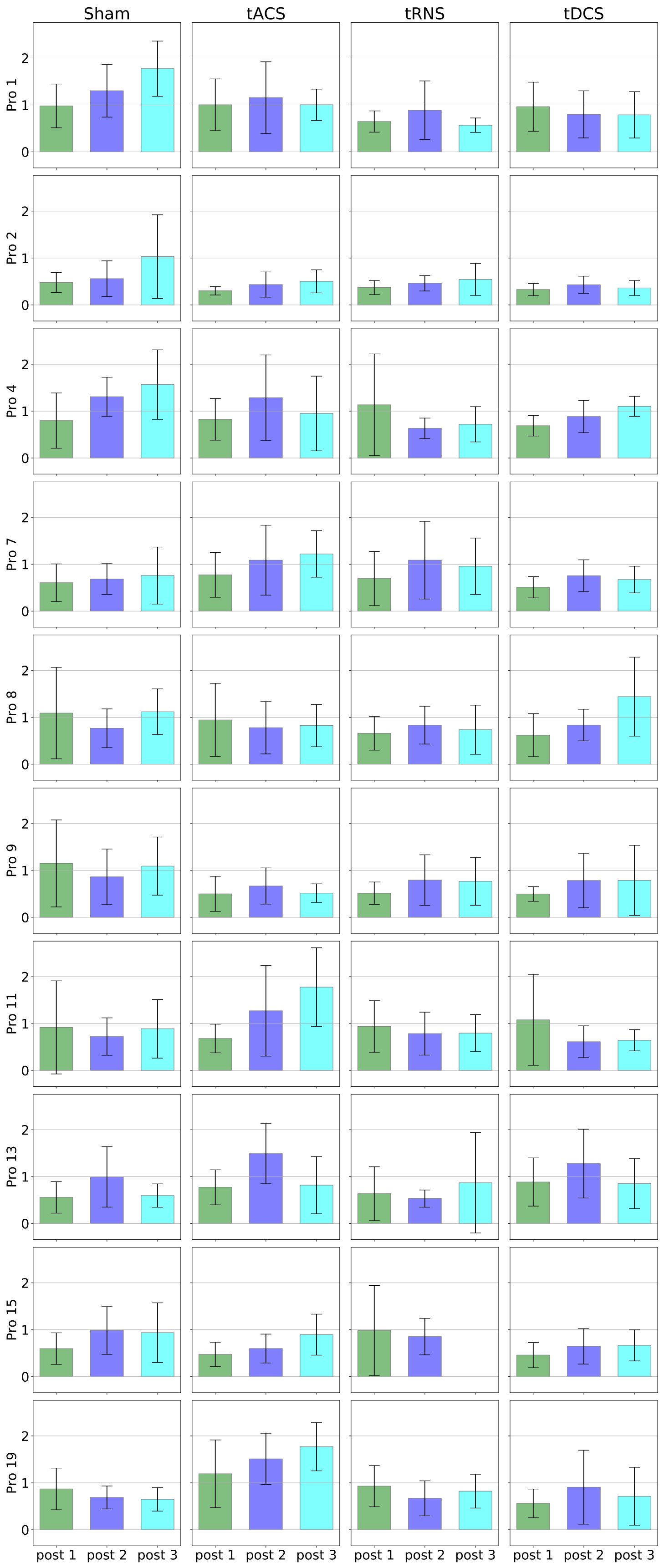

Figure S4: Individual-level difference measured by ProMPs and *symmetric KL-divergence* on 10 participants. Each bar shows the mean and standard deviation averaged over 8 experiments and inward/outward movements. The missing bars correspond to the filtered outliers or corrupted data.
